# Structure-Dependent Resistance to Plasma Impact and Terahertz Shielding Stability of MXene/Aramid Nanofiber Composite Films

**DOI:** 10.3390/ma19112195

**Published:** 2026-05-22

**Authors:** Yizhou Luo, Jingyu Wang, Xing Luo, Hengpei Su, Zelin Zhao, Wanxia Huang

**Affiliations:** 1 College of Materials Science and Engineering, Sichuan University, Chengdu 610065, China; 2 State Key Laboratory of Electronic Thin Film and Integrated Devices, School of Physics, University of Electronic Science and Technology of China, Chengdu 611731, China

**Keywords:** aramid nanofibers, composite structure, MXene, plasma, terahertz shielding

## Abstract

To improve the durability of terahertz (THz) electromagnetic shielding materials in atomic oxygen environments relevant to low Earth orbit (LEO), two MXene/para-aramid nanofiber (ANF) composite architectures were designed, including a uniformly blended structure and a sandwich configuration. Ti_3_C_2_T_x_ MXene was used as the conductive phase, while ANF served as a protective matrix. Oxygen plasma treatment was employed to simulate atomic oxygen exposure. The results show that the plasma resistance of blended films strongly depends on MXene content. Increasing the MXene fraction enhances conductive network redundancy and reduces conductivity degradation. In contrast, the sandwich-structured film exhibits superior structural stability. The outer ANF layers effectively limit direct plasma–MXene interaction and undergo surface carbonization during plasma exposure, forming an additional diffusion barrier. As a result, the sandwich film maintains stable THz shielding performance, with the average shielding effectiveness increasing from 42.6 dB to 44.9 dB after plasma treatment. These results indicate that structural regulation of the internal conductive network, which limits plasma penetration, is essential for maintaining stable MXene-based THz shielding performance under oxidative plasma conditions.

## 1. Introduction

With the rapid development of 6G air–ground integrated communications, satellite communication systems are facing complex and variable electromagnetic wave interference. The terahertz (THz) frequency band (0.1~10 THz) is considered a potential candidate for 6G communication. Therefore, the development of electromagnetic shielding materials for the terahertz band is of great significance [1,2].

Atomic oxygen (AO) is a major component of the low Earth orbit (LEO) atmosphere, with an altitude range of 200–700 km, and it has a particularly significant impact on satellite communication systems [3]. To achieve lightweight and functional integration, 6G satellite communication devices extensively use polymer dielectric substrates and flexible circuit materials. However, these materials are prone to chemical bond fracture and surface erosion under sustained high-speed atomic oxygen attack [4]. Additionally, atomic oxygen impacts spacecraft at a relative velocity of approximately 8 km/s, with an energy of 5 eV, far exceeding the bond dissociation energy of most polymer materials, making them highly susceptible to physical and chemical degradation on their surfaces [5,6].

MXenes are an emerging class of two-dimensional materials consisting of transition metal carbides, nitrides, or carbonitrides [7]. MXene materials generally exhibit high electrical conductivity, excellent mechanical properties, high specific surface area, and tunable surface activity [8,9,10]. Ti_3_C_2_T_x_ MXene has been proven to possess excellent terahertz shielding performance [11], but it faces challenges in satellite communication systems, where atomic oxygen-induced layer-by-layer oxidation and Ti-C bond fracture under atomic oxygen impact can damage the conductive network, rapidly degrade shielding performance, and even cause structural brittle fracture [12,13,14,15].

Aramid nanofibers (ANF) are one-dimensional nanomaterials composed of poly(p-phenylene terephthalamide) (PPTA). The rich amide and hydrogen bond network between molecular chains provides ANF with exceptional mechanical properties and structural stability [16,17]. Studies have shown that ANF films can achieve tensile strengths above 200 MPa and maintain a fracture elongation of approximately 15%, while remaining structurally stable in a wide temperature range from −196 °C to 300 °C, making them particularly suitable for extreme environments such as aerospace applications [16,17,18,19].

Furthermore, the surface of ANF is rich in functional groups (-NH_2_, -COOH), which provide ideal interface binding sites for composite formation with other materials. Strong interface bonding can be achieved through hydrogen bonding and van der Waals forces [20]. Particularly as a flexible matrix material, ANF not only provides mechanical support but also effectively suppresses the oxidation of functional fillers, enhancing the durability and lifespan of composite materials [19].

In this study, Ti_3_C_2_T_x_ MXene was used as a terahertz shielding conductive material, and para-aramid nanofibers (ANF) were compounded with MXene to form flexible, self-supporting films, in which the ANF acts as a protective material.

For comparison, two types of composite films were constructed: a uniformly blended MXene/ANF film, which represents the homogeneous structure adopted in most reported MXene-based electromagnetic shielding materials, and a sandwich-structured ANF/MXene/ANF film [21,22,23,24]. In the blended structure, plasma can readily penetrate into the interior of the material and oxidize the MXene nanosheets, causing fracture of the conductive network and a consequent rapid decline in shielding performance. To overcome this limitation, the ANF/MXene/ANF sandwich structure was designed, in which the upper and lower dense ANF networks serve as physical barriers that block the diffusion of plasma into the internal MXene conductive layer. The plasma impact mode of a plasma cleaner was employed to simulate atomic oxygen bombardment typical of the near-Earth orbit environment, and the changes in terahertz shielding effectiveness of both composite films upon atomic oxygen exposure, together with the underlying material mechanisms, were systematically investigated. Plasma impact mode from a plasma cleaner is used to simulate atomic oxygen bombardment in the extreme environment of near-Earth orbit, and the changes in the terahertz shielding effectiveness of these two composite films under atomic oxygen exposure, along with their material mechanisms, are investigated.

## 2. Materials and Methods

### 2.1. Materials

All materials used for fabricating MXene and MXene/ANF composite films were used as received without further purification, unless otherwise stated. Ti_3_AlC_2_ MAX phase (600 mesh) from Jilin Yiyi Technology Co., Ltd., Changchun, China. HCl (37 wt%, CP grade) from Chengdu Kelong Chemical Co., Ltd., Chengdu, China. LiF (99%, AR grade) from Aladdin Biochemical Technology Co., Ltd., Shanghai, China. Para-aramid nanofiber (para-ANF) aqueous dispersion (0.05 wt%, nominal properties: average diameter 40–60 nm, average length 0.6–1.8 μm, tensile strength 3880 MPa) from Kuaigou Research Nanomaterials Co., Ltd., Dongguan, China. The 0.05 wt% dispersion was selected because it exhibits excellent colloidal stability without obvious agglomeration, and the film formed by vacuum filtration has optimal mechanical flexibility and surface uniformity.

### 2.2. Preparation of Ti_3_C_2_T_x_ MXene

In this experiment, Ti_3_C_2_T_x_ MXene was prepared using the hydrochloric acid (HCl) hydrothermal method. First, 15 mL of 12 mol/L HCl was added to 5 mL of deionized water, followed by the addition of 1 g of LiF. The mixture was heated in an oil bath at 35 °C for 20 min with continuous stirring, resulting in a 20 mL LiF/HCl etching solution. Then, 1 g of Ti_3_AlC_2_ MAX powder was added to the 20 mL LiF/HCl etching solution, and the mixture was heated at 35 °C in an oil bath for 24 h with stirring. After etching, the solution was transferred into centrifuge tubes and centrifuged at 5000 rpm for 3 min using deionized water until the pH reached above 5. The supernatant was discarded, and 20 mL of deionized water was added to the precipitate. The mixture was then sonicated for 1 h in a conical flask, followed by centrifugation at 3500 rpm for 30 min. The resulting dark green supernatant is the monolayer Ti_3_C_2_T_x_ dispersion.

### 2.3. Preparation of Uniformly Blended ANF/MXene and Sandwich-Structured ANF/MXene/ANF Composite Films

The uniformly blended ANF/MXene composite films were prepared by mixing aramid nanofiber dispersion (1 mg/mL) and MXene dispersion (1 mg/mL) at different mass ratios (6:4, 7:3, and 8:2), followed by stirring at room temperature for 2 h to ensure uniform dispersion. The resulting mixture was then vacuum-filtered to obtain the uniformly blended ANF/MXene composite film.

**Figure 1:**
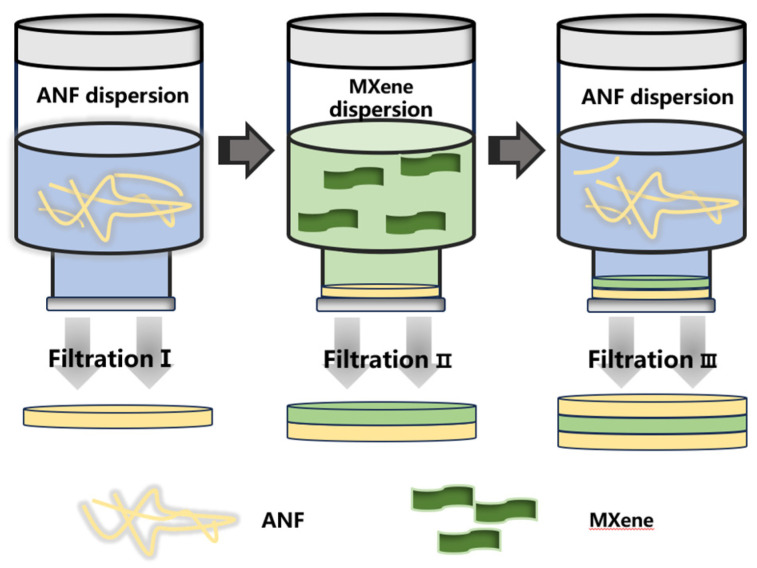
Layer-by-layer vacuum-assisted filtration fabrication route of the ANF/MXene/ANF sandwich-structured composite film, where the sandwich architecture is constructed via three sequential filtration steps of ANF dispersion, MXene dispersion, and ANF dispersion.

The ANF/MXene/ANF sandwich-structured composite film was fabricated via a sequential vacuum-assisted filtration process, as described below (Figure 1). First, the aramid nanofiber dispersion was diluted to 1 mg/mL and stirred until uniformly dispersed. Then, 3 mL of the ANF dispersion was vacuum-filtered to deposit an ANF layer, which served as the bottom substrate. Subsequently, the MXene dispersion was filtered onto the ANF substrate to form a bilayer (MXene/ANF) composite film. Finally, another portion of the ANF dispersion was filtered onto the surface of the MXene layer, yielding the ANF/MXene/ANF sandwich-structured composite film. The thickness of each layer was quantitatively controlled by precisely adjusting the volume and concentration of the suspension. The vacuum level was maintained at 0.08 MPa throughout the entire filtration process, and the filtration time for each layer was fixed at 30 min to ensure an approximately uniform thickness of all layers.

All the as-prepared films were dried in an oven at 60 °C for 2 h to obtain the uniformly blended ANF/MXene and the ANF/MXene/ANF sandwich-structured composite films.

### 2.4. Simulation of Atomic Oxygen Attack Using Plasma Treatment

In this work, an atomic oxygen (AO) attack experiment was conducted using a plasma cleaner (CPC-FM, Huayixing Tech Co., Ltd., Beijing, China) to simulate the AO environment in low Earth orbit (LEO).

In the LEO environment, atomic oxygen impacts the spacecraft surface at a relative velocity of approximately 7.9 km/s, with a single AO impact energy of about 5 eV, leading to direct sputtering and removal of surface atoms [25]. Plasma cleaning technology removes contaminants through the synergistic action of physical bombardment and chemical etching, in which oxygen plasma is the most active species [14].

In the plasma discharge environment, high-energy electrons undergo inelastic collisions with oxygen molecules, generating a large number of highly reactive oxygen plasma species via the electron-impact dissociation reaction (e^−^ + O_2_ → e^−^ + O^−^ + O^−^) [25]. The fast-moving oxygen plasma species can subsequently combine with electrons to form atomic oxygen (e^−^ + O^−^ → O), which is analogous to the generation mechanism of atomic oxygen in actual vacuum environments [26]. Both species reach the material surface with relatively high kinetic energy.

In this study, the physical impact of the plasma was realized by adjusting the oxygen plasma power of the plasma cleaner [27]. The vacuum degree was set to 60 Pa. Under this pressure, gas molecules are relatively dense, the mean free path of ions is short, collisions are very frequent, and the average energy of ions reaching the surface is between 8 and 20 eV. The power was set to 200 W, and the plasma flux was estimated to be on the order of 10^18^–10^19^ ions/(cm^2^·s) based on the discharge current and chamber volume. It should be noted that this flux is 2–3 orders of magnitude higher than that of professional magnetically filtered atomic oxygen plasma sources (10^15^–10^16^ ions/(cm^2^·s)) specifically designed for LEO environment simulation, as reported by Maldonado et al. [28]. Taking the maximum average atomic oxygen flux in the actual space environment as 10^13^ atoms/(cm^2^·s). Nevertheless, the ion energy range (8–20 eV) produced by our experimental setup is highly consistent with the most probable energy range (2.8–7 eV) of atomic oxygen ions in the real LEO environment at altitudes of 275–550 km measured by Maldonado et al. [28] using a retarding potential analyzer. This similarity in energy suggests that the physical bombardment and chemical oxidation mechanisms observed in our experiment remain, in their essential features, representative of the actual space environment. Therefore, although this test cannot be used to predict the absolute service life of materials in space, it can serve as a reasonable basis for comparing the plasma resistance of different composite architectures, which is the core objective of this study. The inherent limitations of the accelerated aging test are further discussed in Section 4.

### 2.5. Electrical Conductivity Measurement

The electrical conductivity of all films before and after plasma treatment was tested by the four-point probe method using an RTS-9 four-probe resistivity meter (Guangzhou Four Probe Technology Co., Ltd., Guangzhou, China).

For each sample, 3 parallel specimens were tested with 5 test points per specimen. The final result was taken as the average of all tests, and the conductivity was calculated from the measured square resistance and the average thickness of the film.

### 2.6. Terahertz Shielding Effectiveness Measurement and Calculation

Terahertz time-domain spectroscopy (THz-TDS) is a coherent detection method. By measuring the time-domain waveform of a terahertz pulse after interaction with a sample and converting it into a frequency-domain spectrum via Fourier transform, the amplitude and phase information are obtained, from which the terahertz shielding performance parameters of the material can be derived.

In this study, a QT-TRS1000 terahertz time-domain spectroscopy system was used to measure the terahertz shielding performance of the as-prepared Ti_3_C_2_T_x_ films.

## 3. Results

### 3.1. Structure and Morphology of Ti_3_C_2_T_x_ MXene, Uniformly Blended (MXene/ANF) and Sandwich Structure (ANF/MXene/ANF) Composite Films

**Figure 2:**
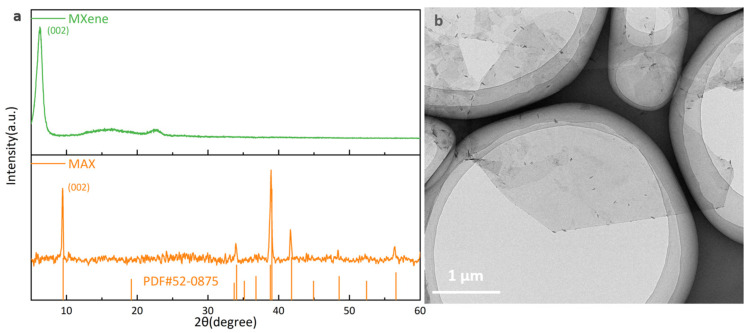
Structural and morphological characterization of the as-prepared Ti_3_C_2_T_x_ MXene. (**a**) X-ray diffraction (XRD) patterns of the pristine Ti_3_AlC_2_ MAX phase precursor and the etched Ti_3_C_2_T_x_ MXene, the (002) diffraction peak shifts to a lower angle after etching, confirming the successful removal of the Al atomic layer; (**b**) transmission electron microscopy (TEM) image of the exfoliated few-layer Ti_3_C_2_T_x_ MXene nanosheets, which exhibits a typical two-dimensional ultrathin structure.

The two-dimensional transition metal carbide Ti_3_C_2_T_x_ MXene is the core functional material in this study, and its intrinsic properties directly determine the electromagnetic shielding effectiveness of the composite films. As shown in Figure 2a, the X-ray diffraction (XRD) pattern reveals that the Al peak at 39° in the Ti_3_AlC_2_ MAX phase has disappeared, and the (002) crystal plane peak shifted to a lower angle. The disappearance of the Al peak at 39° and the shift in the (002) peak confirm Al extraction and interlayer expansion. The TEM image presents a typical two-dimensional lamellar structure (Figure 2b), confirming the successful preparation of two-dimensional Ti_3_C_2_T_x_ MXene.

**Figure 3:**
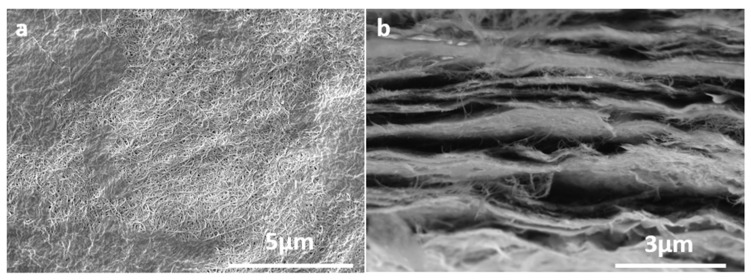
Morphological characterization of the uniformly blended ANF/MXene composite film. (**a**) Surface SEM image of the composite film; (**b**) cross-sectional SEM image of the composite film.

**Figure 4:**
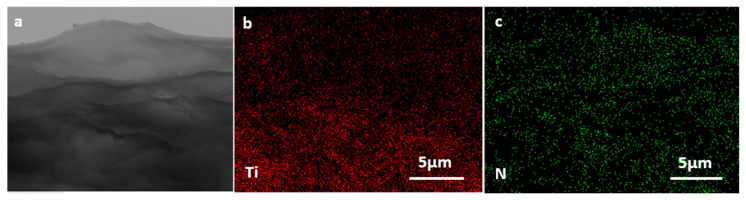
Cross-sectional microstructure and elemental distribution of the uniformly blended ANF/MXene composite film. (**a**) Low-magnification cross-sectional SEM image showing the typical lamellar stacking structure formed by the interpenetration of MXene nanosheets and ANF; (**b**) EDS elemental mapping of Ti (characteristic element of Ti_3_C_2_T_x_ MXene); (**c**) EDS elemental mapping of N (characteristic element of ANF).

Figure 3a shows the surface morphology of the blended ANF/MXene composite film, where both two-dimensional MXene and one-dimensional ANF can be observed on the surface. Figure 3b presents the cross-sectional SEM morphology of the blended ANF/MXene composite film, revealing a typical MXene lamellar stacking structure, with an ANF network distributed between the layers. As shown in Figure 4a, the uniformly blended ANF/MXene composite film exhibits a lamellar stacking structure along the thickness direction. MXene and ANF are sequentially deposited on the filter membrane under vacuum, forming an alternating stacked structure. Figure 4b shows the elemental distribution map of Ti (red signal) from the cross-sectional SEM image. As the characteristic element of Ti_3_C_2_T_x_ MXene, Ti exhibits a relatively uniform full coverage across the entire cross-section, indicating that MXene can form a continuous network in the uniformly blended composite system. Figure 4c presents the elemental distribution map of N (green signal), a characteristic element of ANF, where N is uniformly distributed without noticeable phase separation or localized agglomeration. This confirms that ANF is uniformly intercalated within the gaps of the MXene sheets, providing further validation that the preparation process in this study successfully achieved a uniform blend of the two components.

**Figure 5:**
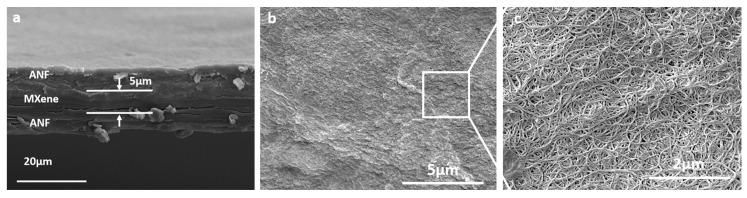
Morphological characterization of the ANF/MXene/ANF sandwich-structured composite film. (**a**) Cross-sectional SEM image of the sandwich-structured film, showing a distinct three-layer structure with an intermediate MXene layer; (**b**) low-magnification surface SEM image of the sandwich-structured film; (**c**) high-magnification surface SEM image of the sandwich-structured film.

Figure 5a shows the surface and cross-sectional morphological characteristics of the ANF/MXene/ANF sandwich structure composite film. From the cross-sectional image, it is evident that the sandwich structure of the ANF/MXene/ANF composite film exhibits a distinct layered architecture, with the MXene conductive layer and the ANF protective layers tightly bonded, showing good interface adhesion. The slight thickness fluctuation of the ANF layers observed in Figure 5a is caused by minor differences in the local flow rate of the suspension during filtration. This fluctuation is within the experimental error range and has no significant effect on the overall mechanical properties and terahertz shielding performance of the composite films. As seen in Figure 5b, the surface layer of the ANF/MXene/ANF sandwich structure composite film consists of a dense layer formed by stacked ANF, with the fibrous ANF tightly wound, providing plasma impact protection for the conductive MXene layer.

### 3.2. Effect of Plasma Treatment on the Surface Morphology and Chemical Structure of Pure MXene Films, Uniformly Blended (MXene/ANF), and Sandwich Structure (ANF/MXene/ANF) Composite Films

**Figure 6:**
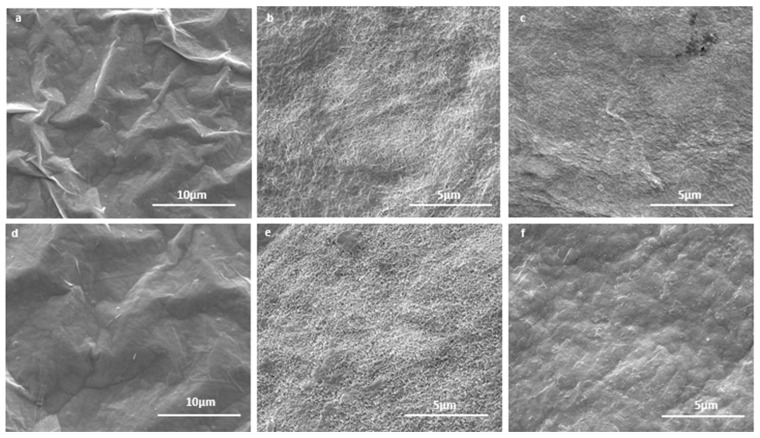
Surface SEM images of the different films before and after plasma treatment. (**a**,**d**) Pure Ti_3_C_2_T_x_ MXene film; (**b**,**e**) uniformly blended ANF/MXene composite film; (**c**,**f**) ANF/MXene/ANF sandwich-structured composite film; (**a**–**c**) before plasma treatment, (**d**–**f**) after 4 h plasma treatment.

The surface microstructure of pure MXene films before and after plasma impact is shown in Figure 6a,c. Before impact, the MXene film exhibited complete and continuous layers with no obvious defects, and the overall structure was dense and flat, providing a solid structural foundation for the construction of a continuous conductive network and high electromagnetic shielding performance. After 4 h of plasma treatment, the surface morphology of the MXene film changed: the originally clear and sharp wrinkles on the sheets became noticeably passivated and blurred, with a significant increase in the overall roughness of the film.

The uniformly blended ANF/MXene composite film (shown in Figure 6b,e) showed noticeable damage on the surface after plasma impact treatment. The originally continuous ANF network was fractured, exposing broken MXene sheets, and a large number of irregular protruding particles formed on the surface, leading to a significant increase in roughness. This was due to the direct plasma impact on the MXene sheets on the film’s surface, while the ANF network was also damaged by the impact. This explains the reduction in conductivity and terahertz shielding effectiveness of the uniformly blended film after plasma exposure.

The ANF/MXene/ANF sandwich structure composite film is shown in Figure 6c,f. After plasma impact, the surface layer of ANF experienced fractures and some carbonized particles formed, but there was no exposure of the MXene sheets or structural damage.

**Figure 7:**
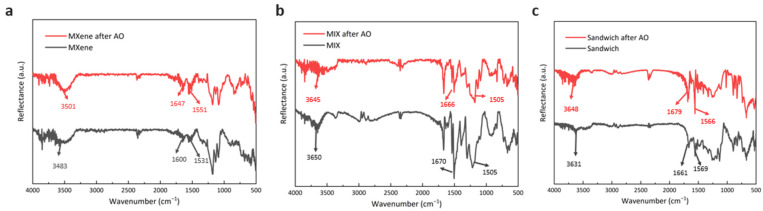
FTIR spectra of the different films before and after plasma treatment. (**a**) Pure Ti_3_C_2_T_x_ MXene film; (**b**) uniformly blended ANF/MXene composite film; (**c**) ANF/MXene/ANF sandwich-structured composite film. Black lines: before plasma treatment; red lines: after plasma treatment.

Figure 7 compares the infrared reflection spectra of MXene films and uniformly blended ANF/MXene composite films before and after plasma impact. According to the latest MXene FTIR spectral library established by the Gogotsi team [29], the FTIR spectrum of Ti_3_C_2_T_x_ MXene is mainly divided into two regions: the 4000–1400 cm^−1^ region corresponds to the vibrations of interlayer water and surface functional groups, while the 800–400 cm^−1^ region is the characteristic fingerprint area for the MXene framework’s Ti–C/Ti–O vibrations.

From an overall trend, the MXene film before plasma bombardment maintains a high reflectance over a broad wavenumber range, exhibiting typical optical behavior of metallic conductive materials. This is closely related to the intrinsic high conductivity and intact lattice structure of MXene. After plasma impact, the MXene film shows a characteristic –OH reflection peak at 3501 cm^−1^, which is blue-shifted compared to the –OH stretching vibration peak (3483 cm^−1^) in MXene. This shift indicates that the hydrogen bond network has been partially disrupted, weakening the binding of the –OH group and increasing its vibrational frequency [24]. The enhancement near 1647 cm^−1^ corresponds to the C=O stretching vibration and the bending vibration of adsorbed water, indicating that the plasma treatment induced the formation of new oxygen-containing functional groups on the MXene surface [9,30]. Meanwhile, new peaks appear in the range of 700–750 cm^−1^, which are attributed to the Ti–O–Ti vibration characteristic of the anatase phase of TiO_2_ [31]. This indicates that the oxidation after plasma treatment is not a simple surface passivation, but rather the formation of an oxide layer with a certain degree of crystallinity. The spectral evolution indicates that plasma exposure initially involves reactions with surface functional groups, followed by progressive oxidation of the Ti–C framework [32]. Subsequently, it gradually erodes the Ti–C lattice of MXene, causing the cleavage of Ti–C bonds and the formation of Ti–O bonds, ultimately resulting in the generation of a TiO_2_-dominated oxide layer on the surface.

Figure 7b shows a comparison of the infrared reflectance spectra of the uniformly blended ANF/MXene composite film before and after plasma treatment. The peaks at 3360 and 1666 cm^−1^ correspond to the stretching vibrations of N–H and C=O [33]. The C=O stretching band shifts from 1600 cm^−1^ to 1666 cm^−1^ after compositing MXene with ANF, suggesting a reconstruction of the hydrogen-bonding environment at the interface. Such blue-shift behavior is commonly associated with strong intermolecular hydrogen bonding between polymer amide groups and surface –OH/–O terminations of MXene nanosheets [34]. After plasma treatment, the N–H stretching vibration (3360 cm^−1^) is significantly weakened, suggesting that plasma attack causes the cleavage or oxidation of N–H bonds in the ANF molecular chains. This is because the dissociation energy of the N–H bond is relatively low, making it susceptible to fracture upon impact by high-energy atomic oxygen, thereby disrupting the integrity of amide groups and the regular arrangement of molecular chains [35,36]. However, the carbonaceous structure formed after the disruption remains densely distributed on the surface of the MXene matrix, preventing further damage to the interior.

Figure 7c presents the FTIR reflectance spectra of the ANF/MXene/ANF sandwich-structured film before and after plasma treatment. After plasma exposure, an increase in absorption intensity is observed in the 3400–3200 cm^−1^ region, accompanied by band broadening. This enhancement suggests the formation of additional hydroxyl groups and the reorganization of hydrogen-bonding interactions within the outer ANF layer, indicating surface oxidation and modification of the hydrogen-bond network.

The aliphatic C–H stretching region (2950–2850 cm^−1^) exhibits negligible variation after plasma treatment, implying that the aromatic backbone of ANF remains largely intact and that degradation is confined primarily to surface functional groups rather than extensive chain scission [37].

A pronounced increase in intensity is observed near 1700 cm^−1^, corresponding to C=O stretching vibrations [38,39]. The strengthened carbonyl-related absorption confirms the occurrence of surface oxidation reactions induced by plasma exposure [40]. Meanwhile, the amide II band around 1540 cm^−1^ shows only minor changes, suggesting that the bulk amide structure of ANF is not significantly destroyed [41].

In the 1300–1000 cm^−1^ region, multiple absorption bands become more intense and complex after plasma treatment. These changes are associated with C–O and C–O–C stretching vibrations, further supporting the introduction of oxygen-containing functional groups on the film surface. Importantly, no additional Ti–O-related absorption bands are detected in the low-wavenumber region, indicating that the inner MXene conductive layer remains effectively protected from plasma-induced oxidation.

Collectively, these spectral results confirm that plasma-induced chemical modification is confined to the outer ANF layer, while the internal MXene layer maintains structural and chemical integrity, consistent with the proposed dual-barrier protection mechanism of the sandwich architecture.

### 3.3. Effect of Plasma Treatment on the Terahertz Shielding Performance of the MXene Film and Two Types of Composite Films

**Figure 8:**
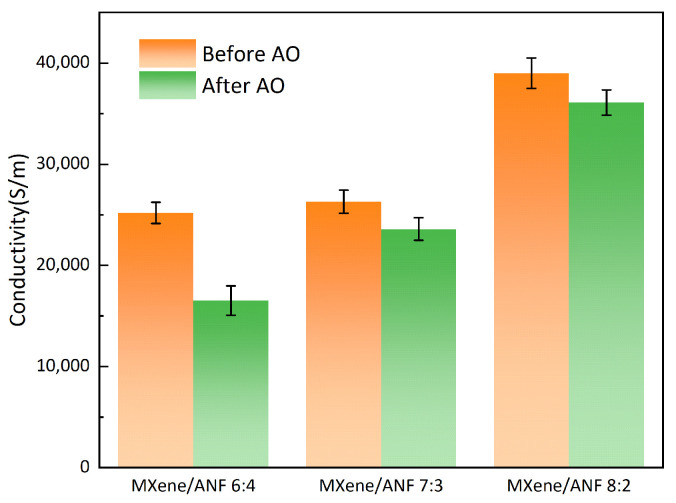
Electrical conductivity of uniformly blended ANF/MXene composite films with different mass ratios (6:4, 7:3, 8:2) before and after plasma treatment.

As shown in Figure 8, the initial electrical conductivity of the uniformly blended ANF/MXene composite films increases significantly with increasing MXene content. This is because a higher content of the conductive MXene phase enables the construction of a more continuous conductive network, thereby enhancing the electron transport capability of the composite system and providing a conductive structural basis for its excellent terahertz electromagnetic shielding performance.

After plasma treatment, the electrical conductivity of the composite films with different mixing ratios shows varying degrees of attenuation, reflecting that the high-energy physical impact and chemical oxidation of the plasma cause certain damage to the conductive network of the composite films. Among them, the MXene/ANF = 6:4 sample exhibits the largest conductivity attenuation, with a reduction of 35.3%. The MXene/ANF = 8:2 sample shows the best stability, maintaining a high electrical conductivity of 36,700 S/m after treatment, with an attenuation of only 6.6%. For the high MXene content sample (MXene/ANF = 8:2), the dense lamellar stacking of MXene constructs a conductive network with high percolation redundancy. Even if the surface MXene nanosheets are oxidized and damaged by plasma erosion, the internal continuous electron transport pathways remain intact, thus maintaining a high conductivity level with only 6.6% attenuation.

**Figure 9:**
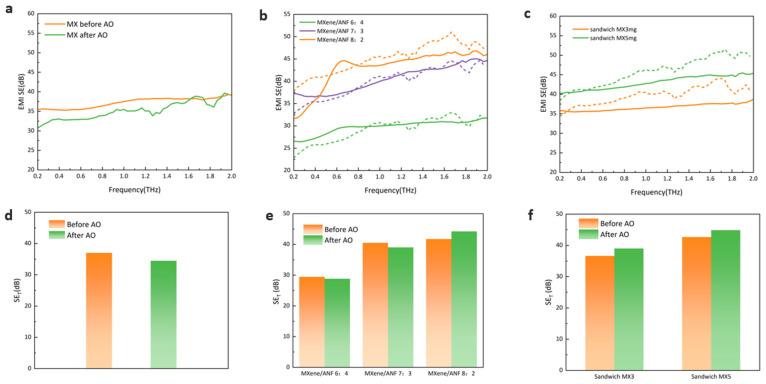
Terahertz EMI shielding performance of the as-prepared films before and after plasma treatment. (**a**) EMI SE spectra of pure MXene film before and after treatment; (**b**) EMI SE spectra of uniformly blended ANF/MXene films with different mass ratios before treatment (solid lines); after plasma treatment (dashed lines); (**c**) EMI SE spectra of sandwich-structured films before treatment (solid lines); after plasma treatment (dashed lines); (**d**–**f**) Average EMI SE values of (**d**) pure MXene, (**e**) uniformly blended films, and (**f**) sandwich-structured films before and after treatment.

The change in shielding performance of the uniformly blended ANF/MXene composite films is consistent with the evolution of electrical conductivity, as described below (Figure 9b,e). For the low-MXene-content samples (MXene/ANF = 6:4 and 7:3), a slight SE attenuation of 1.0–2.0 dB in shielding effectiveness was observed. This attenuation is directly related to the decrease in electrical conductivity of the films after plasma treatment: structural damage to the conductive network reduces the material’s ability to dissipate energy via conductive loss, ultimately leading to a decline in shielding effectiveness. In contrast, the MXene/ANF = 8:2 sample exhibits an increase in shielding effectiveness from 41.8 dB to 44.1 dB. This is attributed to the fact that the conductivity attenuation of this sample is only 6.6%, so the integrity of the core conductive network is largely preserved, and the intrinsic conductive loss capability of the material does not decrease significantly. Additionally, the moderate etching of the sample surface by the plasma increases the surface roughness, which enhances multiple reflection losses of electromagnetic waves, while the small amount of non-conductive phases, such as TiO_2_ formed on the MXene surface, can introduce interfacial polarization. The combined effect of these factors leads to the observed increase in shielding effectiveness.

As shown in Figure 9c,f, the ANF/MXene/ANF sandwich-structured composite film exhibits superior stability after plasma treatment, with its average terahertz shielding effectiveness increasing from 42.6 dB before treatment to 44.9 dB. This result fully confirms the protection mechanism of the sandwich structure: the outer ANF layer serves as a sacrificial precursor, undergoing in situ carbonization under atomic oxygen bombardment to form a continuous carbonaceous layer that acts as a secondary physical barrier against plasma erosion. Meanwhile, the hydrogen bonding network between the ANF and MXene layers maintains the structural integrity of the film, preventing plasma from penetrating along the interlayers and protecting the internal MXene conductive network.

As shown in Figure 9a,d, for the pure MXene film, the THz electromagnetic shielding effectiveness before and after plasma treatment is 37.1 dB and 34.5 dB, respectively, exhibiting a certain degree of attenuation. This indicates that although the pure MXene film itself has some tolerance to atomic oxygen erosion, it is still attacked by atomic oxygen, resulting in a decrease in shielding effectiveness. This can be attributed to the abundant functional groups on the MXene surface, which may form an oxide layer during exposure, thereby preventing further erosion to some extent.

In summary, the uniformly blended ANF/MXene composite film can improve the stability of the pure MXene film, whereas the ANF/MXene/ANF sandwich-structured composite film protects the internal MXene conductive layer through the physical barrier effect of the outer ANF layer, the structural stabilization effect of the interfacial hydrogen bonding, and the secondary protection of the carbonaceous layer. This endows the composite film with excellent shielding performance and stability under plasma treatment, providing a new strategy for the design of high-performance terahertz electromagnetic shielding materials for use in the low Earth orbit space environment.

## 4. Discussion

Plasma exposure affects MXene-based films through both chemical reactivity and structural factors. The way reactive species move within the film plays an important role in determining how oxidation progresses and how shielding performance changes.

For pure Ti_3_C_2_T_x_ films, the stacked sheets expose surface terminations directly to incoming plasma species. Oxidation starts at these surface sites and extends into the Ti–C framework, accompanied by TiO_2_ formation. Because electrical transport depends on continuous contact between adjacent nanosheets, even partial surface oxidation interrupts charge transfer. Under these conditions, degradation is dominated by surface reactions, and oxygen can gradually penetrate along the interlayer pathways.

In the uniformly blended MXene/ANF films, the ANF network modifies how reactive species access the conductive phase. The intercalated nanofibers partially cover MXene sheets, but their distribution is not perfectly homogeneous. Some regions therefore remain insufficiently protected and become preferential oxidation sites. This behavior is consistent with the larger conductivity loss observed in samples with lower MXene content. In these films, shielding stability is mainly sustained by conductive network redundancy rather than by effective blocking of oxygen diffusion.

The sandwich configuration presents a different situation. The outer ANF layer separates the MXene layer from direct plasma exposure and increases the diffusion distance for reactive oxygen species. After plasma treatment, the formation of a carbon-rich surface further reduces oxygen permeability. Oxidation is therefore largely confined to the outer region, and the inner MXene layer retains structural continuity. Compared with the other architectures, the degradation rate in this structure appears to be limited by oxygen transport rather than by immediate surface reaction.

The slight increase in terahertz shielding effectiveness after plasma treatment in high-MXene-content and sandwich samples can be explained by two concurrent effects. The conductive network remains largely intact, preserving reflection loss, while moderate surface oxidation introduces additional interfaces and roughness. These structural changes promote multiple reflection and interfacial polarization, contributing to energy dissipation. The final shielding performance reflects the balance between conductive continuity and newly introduced dielectric loss.

Taken together, the results show that plasma resistance in MXene-based shielding films depends strongly on structural control of oxygen transport. Designing architectures that restrict diffusion while preserving conductive pathways is key to maintaining stable terahertz shielding under oxidative conditions.

While the sandwich-structured films clearly show improved plasma resistance, we should keep in mind that our accelerated plasma test differs in important ways from the real low Earth orbit (LEO) environment, as discussed by Kleiman et al. [28]. In our experiment, the atomic oxygen flux was about 10^18^–10^19^ ions/(cm^2^·s), which is 10^5^–10^6^ times higher than the typical flux experienced in LEO (around 10^13^ atoms/(cm^2^·s)). Because the oxidation happens so quickly, the reaction paths and kinetics may not fully match the slow, continuous erosion that materials undergo in orbit. In a real space environment, there is enough time for a stable passivation layer to develop on the surface, whereas the intense, short-duration plasma we used could trigger non-equilibrium reactions and local heating effects that would not normally occur. For this reason, the long-term behavior of these films under actual space conditions still needs to be confirmed.

Another point worth noting is that the LEO environment is not just about atomic oxygen. It also involves high vacuum, ultraviolet (UV) radiation, and thermal cycling, all of which can work together to degrade materials [28]. Previous work has shown that UV exposure can weaken polymer chains and make them more vulnerable to oxidation, while repeated temperature swings can introduce thermal stresses and microcracks, speeding up the overall damage. Our study only looked at atomic oxygen alone, and the combined effects of these factors on the shielding stability of MXene/ANF films remain an open question for future research.

That said, the results do offer useful insight: controlling the internal structure to restrict oxygen transport is an effective way to improve the plasma resistance of MXene-based shielding materials. The comparison between blended and sandwich architectures gives a practical starting point for designing more robust aerospace shielding films. Moving forward, we plan to upgrade our experimental setup to better simulate the multi-factor LEO environment and to carry out longer-term ground tests and, eventually, space-flight experiments to gain a more realistic picture of how these composite films perform in service.

## 5. Conclusions

In this work, two MXene/ANF composite architectures were constructed to investigate structure-dependent plasma resistance and terahertz shielding stability under oxygen plasma irradiation.

Pure Ti_3_C_2_T_x_ MXene films suffer from progressive Ti-C bond cleavage and TiO_2_ formation under plasma irradiation, resulting in conductive network disruption and THz shielding performance attenuation. In uniformly blended films, ANF moderates oxidation kinetics but does not prevent direct plasma–MXene interaction, resulting in limited conductivity retention.

In contrast, the ANF/MXene/ANF sandwich architecture fundamentally alters the oxidation pathway through a hierarchical dual-barrier mechanism. The outer ANF layer dissipates kinetic energy of plasma species, while the in situ carbonized surface acts as a secondary diffusion barrier, confining oxidation to the sacrificial region. Consequently, the conductive MXene core remains structurally intact. This rational structural design enables stable and even enhanced terahertz shielding performance after plasma exposure. The results demonstrate that structural regulation of reactive species transport is a key strategy for improving the durability of MXene-based electromagnetic shielding materials in extreme oxidative environments.

## Data Availability

The original contributions presented in this study are included in the article. Further inquiries can be directed to the corresponding author.
